# An Automatic Diagnosis Method of Facial Acne Vulgaris Based on Convolutional Neural Network

**DOI:** 10.1038/s41598-018-24204-6

**Published:** 2018-04-11

**Authors:** Xiaolei Shen, Jiachi Zhang, Chenjun Yan, Hong Zhou

**Affiliations:** Key Laboratory for Biomedical Engineering of Ministry of Education, Zhejiang University, HangZhou, 310027 China

## Abstract

In this paper, we present a new automatic diagnosis method for facial acne vulgaris which is based on convolutional neural networks (CNNs). To overcome the shortcomings of previous methods which were the inability to classify enough types of acne vulgaris. The core of our method is to extract features of images based on CNNs and achieve classification by classifier. A binary-classifier of skin-and-non-skin is used to detect skin area and a seven-classifier is used to achieve the classification task of facial acne vulgaris and healthy skin. In the experiments, we compare the effectiveness of our CNN and the VGG16 neural network which is pre-trained on the ImageNet data set. We use a ROC curve to evaluate the performance of binary-classifier and use a normalized confusion matrix to evaluate the performance of seven-classifier. The results of our experiments show that the pre-trained VGG16 neural network is effective in extracting features from facial acne vulgaris images. And the features are very useful for the follow-up classifiers. Finally, we try applying the classifiers both based on the pre-trained VGG16 neural network to assist doctors in facial acne vulgaris diagnosis.

## Introduction

Acne is a chronic skin disease whose characteristics and mechanisms are very complex. Because of its diversity, we cannot find a unified classification method to assist in diagnosis. In this article, we adopt a type of classification consisting of non-inflammatory lesions (open comedones called blackheads and closed comedones called whiteheads), inflammatory lesions (papules and pustules) and relatively more severe types, nodules and cysts^1^. The data sets and the classifier in this article are all designed to detect these six types of acne vulgaris (blackheads, whiteheads, papules, pustules, modules and cysts) mentioned above on human faces. A patient’s face usually suffers from multiple types of acne lesions at the same time. The causes and treatments of different acne lesions are different, so it is necessary to make an accurate and objective diagnosis for the face with acne vulgaris before treatment^2^. The manual observation and counting acne is the traditional diagnosis method. However, the method is not effective enough. It is labor-intensive, time-consuming and subjective, because the diagnosis results depend on expert’s experience and ability. Therefore, a computerized method is very necessary. Many researches have been done on the automatic diagnosis for facial acne with image processing techniques and machine learning theories.

Automatic diagnosis of facial acne needs to detect and classify the region of interest(ROI). The detection of ROI is mainly equal to the detection of facial skin. Previous automatic diagnosis methods also include specific positioning for various acne. Common skin detection models are based on special color spaces to complete, such as RGB, HSV, YCbCr^3–7^. Due to mixing of chrominance and luminance data, RGB is not a good choice for skin detection. Although YCbCr avoids this problem, its actual detection effect is still unstable and susceptible to some environmental influences^8^. In past automatic diagnosis methods, specific positioning of the acne area is necessary. Chantharaphaichit *et al*. proposed a facial acne area detection method based on grayscale and HSV color space^9^. However, with this method, the color, shape and lighting conditions of acne have a great influence on the detection result. Kittigul and Uyyanonvara proposed a different method based on Heat-Mapping and adaptive thresholding^10^. However, the detection results still contain some noise, such as the area of mouth and skin. The above methods are based on special color spaces and thresholds to achieve the detection of acne area. However, the defects of these methods are criticized for their high dependency on threshold values and lack of generalization ability.

Previous automatic diagnosis methods of acne are mostly based on feature extraction to achieve classification of acne. Chang and Liao, and Malik *et al*. proposed two similar approaches both based on support vector machine and feature extraction to achieve the classification^11,12^. Furthermore, Malik *et al*. classified the severity levels into mild, moderate, severe and very severe. Because of the limitation of classification, these methods can’t achieve the integral analysis with a detailed description.

CNNs have been widely applied to image classification. Sometimes it presents a high recognition ability and gives a better performance than human beings in certain projects, such as the recognition of traffic signs, faces and handwritten digits^13–15^. In recent years, with a lot of research work on the ImageNet data set, the image recognition based on CNN has been continuously improved. In recent studies, CNN with a deeper and wider structure has a large number of parameters. So, the neural network is easy to over fit in the training process. To prevent over fitting, Hinton *et al*. proposed the “dropout” method and the data augmentation method^16–18^.

In this paper, we propose a novel automatic diagnosis method to overcome the shortcoming of classification categories in the previous methods and achieve a holistic analysis of facial acne vulgaris with a detailed description. Different from the old methods, our method extracts features by CNN instead of manual selection. With the novel method, more and deeper features are extracted to enhance classification accuracy and add more classification types^19,20^. The major work in this paper is to achieve the detection of facial skin and the seven-type-classification of facial acne vulgaris by the classifiers with CNN. In the experiment, we compare the validity of extracting features between the CNN model constructed manually by ourselves and the VGG16 model which has been pre-trained on ImageNet. We select the excellent performance model to achieve facial skin area detection and acne seven classification (including 6 types of acne and normal skin) task. On the basis of the classification results, we finally realize the automatic diagnosis and the integral analysis of patients’ faces.

## Proposed Methodology

This prospective study was approved by the institution of DOCTOR MIAO NATIONALITY. All study procedures and dataset were performed in accordance with relevant guidelines and regulations. Informed oral consent for the study and publication was obtained from all patients.

**Figure 1:**
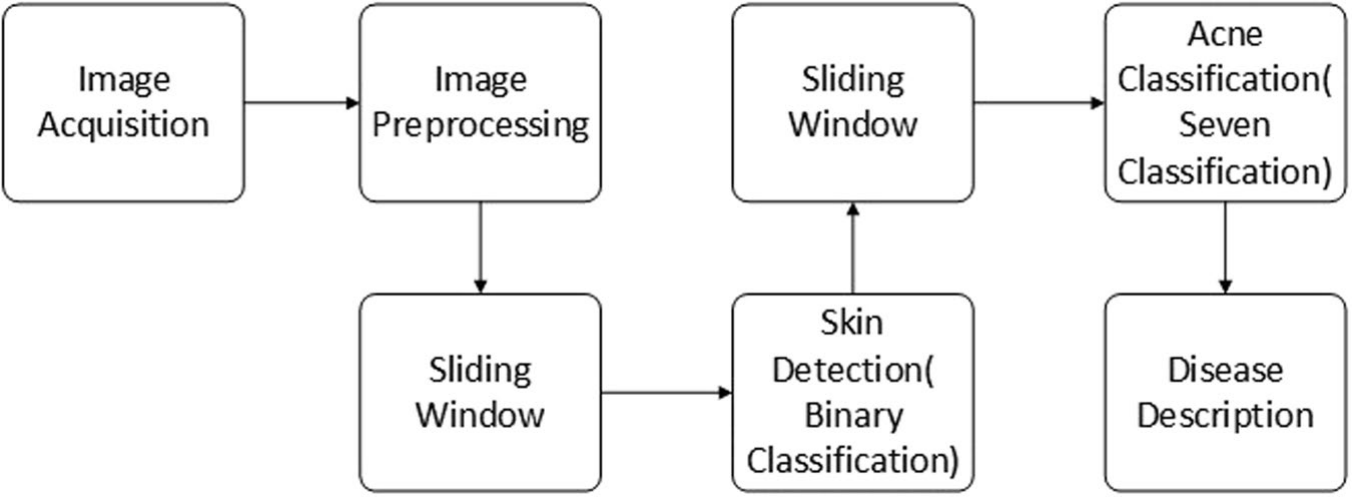
The process of the automatic diagnosis method (sec. 2).

The process of the automatic diagnosis method based on CNN is shown in Fig. 1. There are two main steps: (1) skin detection to locate ROI and (2) seven-type-classification of facial acne vulgaris to achieve the automatic acne diagnosis. The skin detection method is completed by a binary-classifier differentiating skin from non-skin. The binary-classifier with CNN can extract the features from input images and classify them into skin and non-skin to achieve the skin detection. The acne classification method is achieved by a seven-classifier with CNN. The seven-classifier can extract the features from input images by CNN and classify them into one of the seven categories. Then, based on the classification results, integral analysis with a detailed description including acne categories and their respective proportions will be generated.

### The data sets, data augmentation and data preprocessing

**Figure 2:**
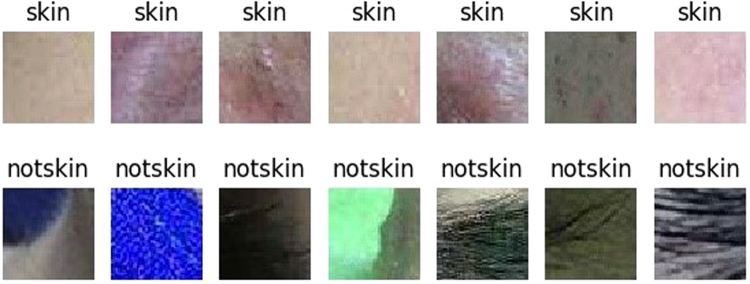
A part of the data set for the training,validation and testing of the binary-classifier (sec. 2.1).

**Figure 3:**
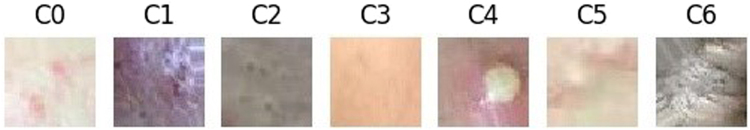
A part of the data set for the training, verification and testing of the seven-classifier (sec. 2.1) Papule (C0), cyst (C1), blackhead (C2), normal skin (C3), pustule (C4), whitehead (C5), nodule (C6).

We collect two data sets for construct a binary-classifier and a seven-classifier respectively. 80% of the each data set is used for training, 10% is used for validation and the rest is used for testing. The data set for the binary-classifier consists of 50 × 50 images which are cut from the original 500 × 500 images manually. This data set contains 3000 skin images and 3000 non-skin images. To analyze facial acne vulgaris, skin images include normal skin and diseased skin. A portion of the data set for the binary-classifier models is shown in the Fig. 2. The data set for the seven-classifier model consists of 50 × 50 images cut from the original 500 × 500 images manually,too. And images augmentation is based on this data set for the seven-classification task. The augmented data set include 6000 blackhead images, 6000 whitehead images, 6000 papule images, 6000 pustule images, 6000 cyst images, 6000 nodule images, 6000 normal skin images. A portion of the data set for the seven-classifier model is shown in the Fig. 3. Furthermore, we also apply the integrated models on 500 × 500 images to evaluate the practical value. The result is shown in section experiments and results.

The original data of seven-classification is so small that it’s very easy to lead the neural network over fitting. We use the simplest and most commonly used method data augmentation to prevent over fitting^17^. In this paper, we used the transformation method of random rotation, shift, shear, scaling and horizontal flip to enlarge our data sets. Transformation can be represented by a transformation matrix. There is a transformation matrix *M*,

1 $$M=[\begin{array}{ccc}A & B & C\\ D & E & F\\ G & H & I\end{array}]$$

*A* and *E* control the scaling of the image, *C* and *F* control the shift of the image, *B* and *D* control the shear of the image. Detailedly, the transform matrix of rotation is

2 $$M=[\begin{array}{ccc}cos\theta  & -sin\theta  & -cos\theta \cdot \frac{h+1}{2}+sin\theta \cdot \frac{w+1}{2}+\frac{h+1}{2}\\ sin\theta  & cos\theta  & -sin\theta \cdot \frac{h+1}{2}-cos\theta \cdot \frac{w+1}{2}+\frac{w+1}{2}\\ 0 & 0 & 1\end{array}]$$

In the matrix, *h* is the height of the picture, *w* is the width of the picture, *θ* is the angle of rotation. The transform matrix of shift is

3 $$M=[\begin{array}{ccc}1 & 0 & tx\\ 0 & 1 & ty\\ 0 & 0 & 1\end{array}]$$

In the matrix, *tx* is the shift size of height, *ty* is the shift size of width. The transform matrix of shear is

4 $$M=[\begin{array}{ccc}1 & -sin(shear) & -zx\cdot \frac{h+1}{2}+\frac{h+1}{2}\\ 0 & cos(shear) & -\frac{h+1}{2}-cos(shear)\cdot \frac{w+1}{2}+\frac{w+1}{2}\\ 0 & 0 & 1\end{array}]$$

In the matrix, shear is the transformation intensity of shear, *h* is the height of the picture, *w* is the width of the picture. The transform matrix of zoom is

5 $$M=[\begin{array}{ccc}zx & 0 & -zx\cdot \frac{h+1}{2}+\frac{h+1}{2}\\ 0 & zy & -zy\cdot \frac{w+1}{2}-\frac{w+1}{2}\\ 0 & 0 & 1\end{array}]$$

**Figure 4:**
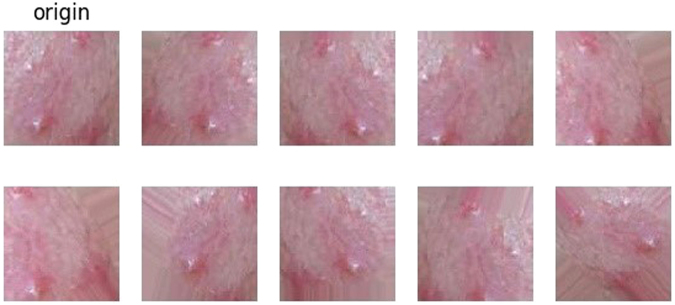
The examples of transformation (sec. 2.1).

In the matrix, *zx* and *zy* control the zoom of the image. Moreover, we set the random horizontal flip image. The examples of transformation are shown in the Fig. 4.

Every original image has been transformed several times, but retained its key features. Additionally, the raw pixels of the images are located between 0–255. They already have a certain standard ability. In the experiment, we used a normalization approach to scale the pixel data, which is beneficial for the gradient descent in the training process.

### Export the feature vector

CNNs have the ability to extract image features. Normally, a deeper CNN can extract more specific and complex features. However, it cannot ensure better performance of the model if it involves too many convolutional layers. In the transfer learning, VGG16 is a more commonly used CNN model. A VGG16 model without top layers is shown in Table 1. On the ImageNet and other large data sets VGG16 has an imposing identifying ability, and its effectiveness of the feature extraction has been proved valid on large data sets^21^. In this paper, we use the pre-trained VGG16 network to extract the characteristics from the binary-classification data sets and the seven-classification data sets, and express their features with a 512-dimension feature vector. The advantage of using pre-trained models is obvious. It not only extracts effective features but also reduces the training time. Furthermore, the validity of extraction feature of pre-trained model has been validated. Relatively speaking, to train a capable network for extracting efficient features needs to consume more resources.

**Table 1:** The pre-trained VGG16 network model without the top layer (sec. 2.2).

Layer	Type of layer
Input (50 × 50 RGB image)	
Block1Conv1	Conv2D-64
Block1Conv2	Conv2D-64
BlockPool	Maxpooling2D
Block2Conv1	Conv2D-128
Block2Conv2	Conv2D-128
Block2Pool	Maxpooling2D
Block3Conv1	Conv2D-256
Block3Conv2	Conv2D-256
Block3Conv3	Conv2D-256
Block3Pool	Maxpooling2D
Block4Conv1	Conv2D-512
Block4Conv2	Conv2D-512
Block4Conv3	Conv2D-512
Block4Pool	Maxpooling2D
Block5Conv1	Conv2D-512
Block5Conv2	Conv2D-512
Block5Conv3	Conv2D-512
Block5Pool	Maxpooling

In this paper, we also construct a CNN. The network structure is shown in Table 2. To train a efficient model for extracting features from the seven-classification data set is more difficult than from the binary-classification data set. So, The model constructed by ourselves is trained, validated and tested on the binary-classification data set, which extract the image features alike VGG16. And we use the pre-trained VGG16 model to extract features from seven-classification data set.

**Table 2:** The convolutional neural network constructed by ourselves (sec. 2.2).

Layer	Type of layer
Input (50 × 50 RGB image)	
Block1Conv1	Conv2D-64
Block1Conv2	Conv2D-64
BlockPool	Maxpooling2D
Block2Conv1	Conv2D-64
Block2Pool	Maxpooling2D
Dropout1	Dropout
Flatten1	Flatten-10816
Dense1	Dense-128
Dropout2	Dropout
Dense2	Dense-2
Soft-max	

### Skin detection (binary-classifier) and acne classification (seven-classifier)

The detection of skin area is based on the binary-classifier. Relatively, to build a binary-classifier with high accuracy is not a difficult task, because the random classification has an accuracy of 50%. The feature vector of the image is extracted by a CNN model, and the classifier is used to classify and output the probability of skin or non-skin to achieve the skin detection. Similarly, the classification of acne is based on the feature extraction by the CNN and classification by the seven-classifier, and output the probability of each acne class. The structure of the binary-classifier is shown in Table 3. Seven-classifier structure is similar to the binary-classifier. The only difference is the 7-dimension output vector at the final dense layer.

**Table 3:** The binary-classifier without CNN for feature extracting (sec. 2.3).

Layer	Type of layer
Input (512 × 1 × 1 feature vector)	
Flatten1	Flatten-512
Dense1	Dense-256
Dropout1	Dropout-256
Dense2	Dense-2
Soft-max	

After training, the seven-type-classifiers can classify six types of acne and healthy skin. Combined with skin-and-non-skin binary-classifier, this classifier can achieve a facial acne diagnosis. This paper introduces a sliding window method to achieve automatic cropping and traversing of the input facial images then classifier of each small area. Statistics of all classification results complete the overall description of facial acne vulgaris.

In this paper, we adopt some techniques to lower the risk of over fitting. We use the “ReLU” activation function and the “dropout” trick to prevent over fitting^17^. Similarly, the data augmentation and the data normalization have the same effect.

## Experiment and Results

### Evaluation method

ROC represents the “Receiver Operating Characteristic” curve, usually used to assess the performance of a binary-classifier. And AUC represents the Area Under ROC. Normally, the classifier’s AUC is larger, the classifier’s performance is better. The straight line connected by point (0,0) and point (1,1) represents a random binary-classifier’s performance^22,23^. The Youden’s index is a summation of the ROC, assessing the validity of the diagnostic marker, and selecting the optimal separation threshold^23,24^. ROC is very appropriate to evaluate the performance of binary-classifier, but less credible to evaluate multi-classifier. So, in this paper, we do not use the extended form of the ROC to evaluate the performance of seven-classifier, we use the normalized confusion matrix as the method to evaluate the performance of seven-classifier. Normalized confusion matrix is normalized from the confusion matrix. Confusion matrix comprises of the numbers of each categories into which the images of our testing data set are classified, including both correct numbers and incorrect numbers^25^.

### Skin detection and the performance of binary-classifier

**Figure 5:**
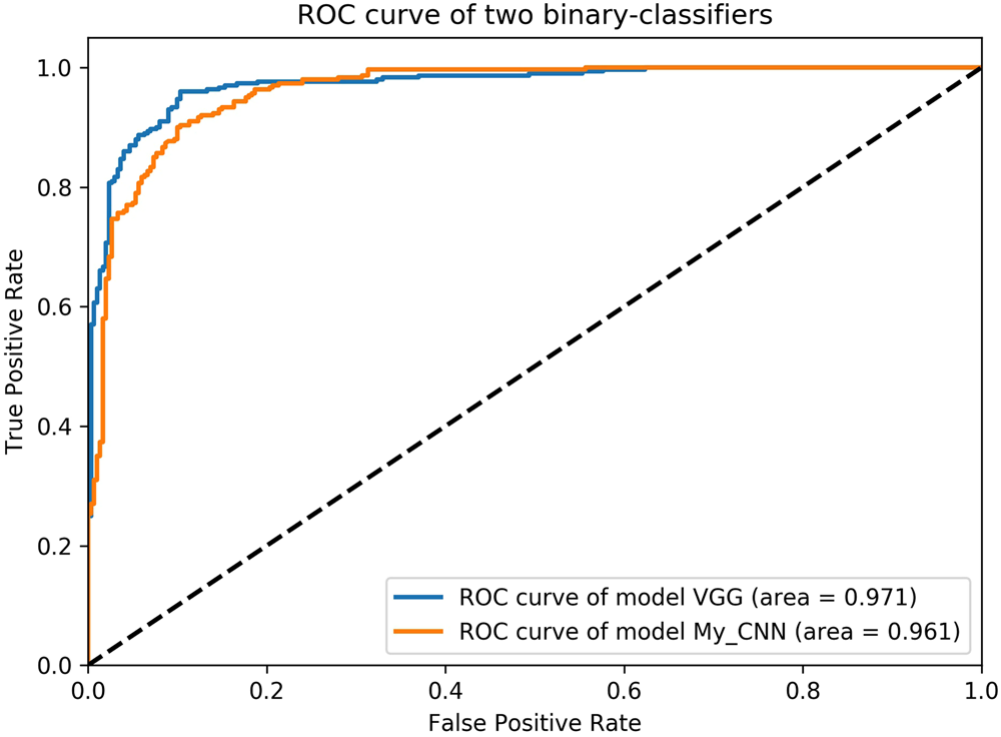
The ROC of two skin-and-non-skin binary-classifier models (sec. 3.2) class 0 represents skin, class 1 represents non-skin. class 0 is regarded as positive example, class 1 is regarded as negative example (epochs = 50). Y-axis is TPR (Sensitivity), X-axis is FPR (1-Specificity).

**Figure 6:**
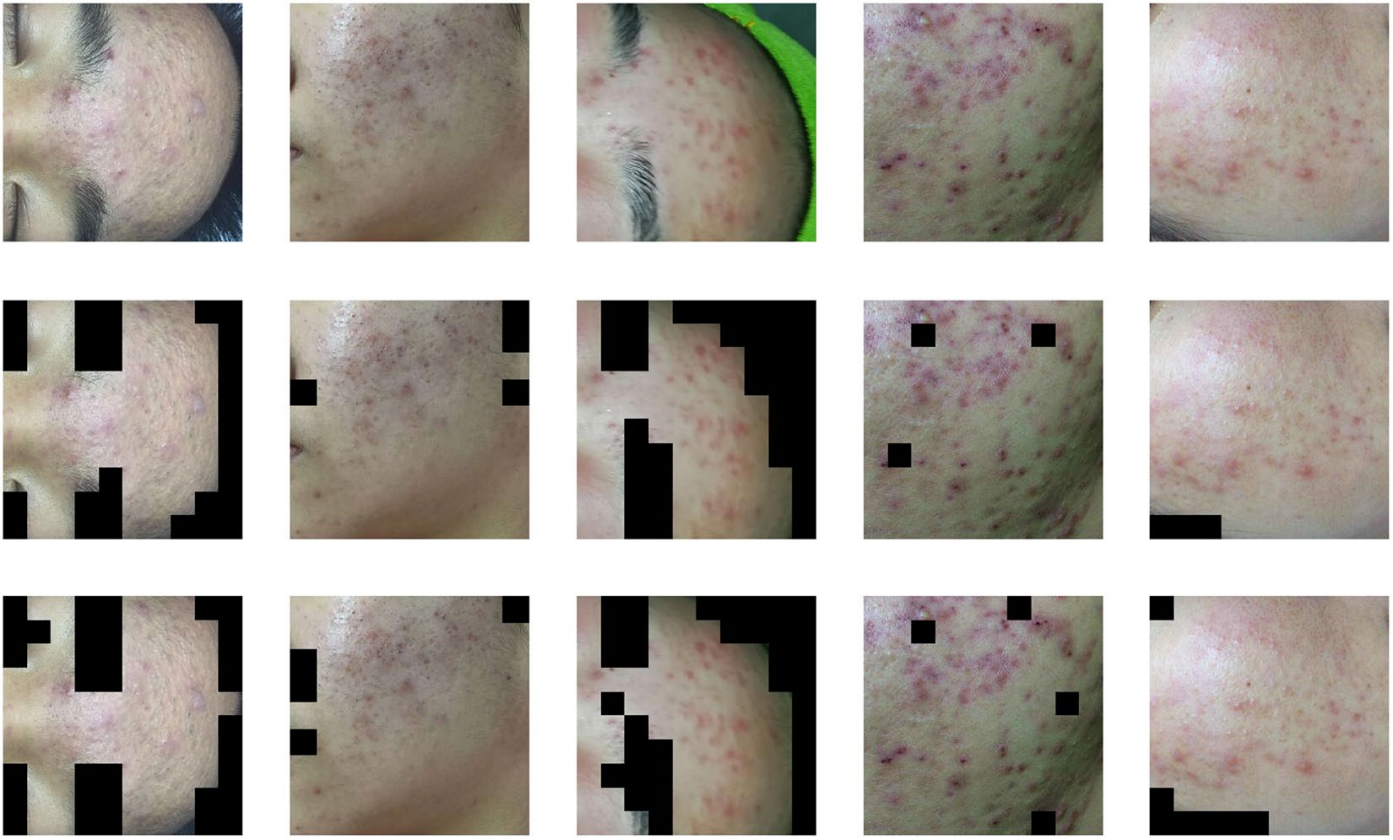
The results of skin detection by the binary-classifier with CNN (sec. 3.2) The first row presents five original images (500 × 500), each of which includes skin areas and non-skin areas. The second row presents five images covered by masks which are built through the binary-classifier based on a manually constructed neural network. The mask is built by 50 × 50 boxes. The third row presents five images covered by masks built through the binary-classifier based on the VGG16 neural network.

Skin detection task is completed by a binary-classifier. In the experiments, we compared the performance of the model constructed by ourselves and the model based on pre-trained VGG16. First of all, we scale the data set to range 0–1 through dividing by 255. When training the model constructed by ourselves, we use the binary cross entropy as the loss function. The binary cross entropy loss is

6 $$L(X,t)=-\,t\,\mathrm{log}\,p(T=\mathrm{1|}X)-\mathrm{(1}-t)\,\mathrm{log}\,p(T=\mathrm{0|}X),$$

where *P*(*T* = *i*|*X*) is the probability that the model assigns to the label *i*, and the binary label *t* ∈ {0,1}. And we use the Adam optimizer whose learning rate is 0.001, beta 1 is 0.9, beta 2 is 0.999 to minimize the loss function. We set the batch size for model’s training is 64, the epochs is 50 and a random seed is 1337 for reproducibility. After 50 epochs, we choose the best model by evaluating the performance of this model on validation data set. When training the model based on pre-trained VGG16, we use the same loss function, optimizer, batch size, epochs and random seed. The difference is that we only need to train the classifier without training the feature extractor. So, its training time is very shorter than the previous model. Similarly, we choose the best model after 50 epochs. What’s more, we tune the feature extractors (the last few convolutional layers) of model based on pre-trained VGG16 with the SGD optimizer whose learning rate is [0.001, 0.01]. However, the performance doesn’t improve obviously. We compare the performance of the two trained models on test data. The test data set hasn’t been seen by the two models. Finally, we will choose the better model to apply into practice. The ROC of the two binary-classifiers are shown in Fig. 5. The detailed index analysis is shown in Table 4 and the results of skin detection(apply into practice) are shown in Fig. 6. The AUC of the model based on pre-trained VGG16 is a little bit bigger. But we choose the model based on pre-trained VGG16 by analyzing the data in Table 4, because it has a good balance between the specificity and the sensitivity. The non-skin areas in the original image including hair, eyes, background, etc. are well detected and covered with black blocks, and the skin areas in the original image are also retained as much as possible. Misjudgments mainly occur when the sliding window contains the junction area of skin area and non-skin. This is the major source of error during detection.

**Table 4:** The performance of the binary-classifier based on VGG16 model and our model (sec. 3.2) Receiver operating characteristic curve (ROC), Area under ROC (AUC), Youden’s index (Y index), Best threshold (Best T), Accuracy (ACC), Sensitivity (SEN), Specificity (SPE) were determined.

Model	Class	AUC	Y index	Best T	ACC	SEN	SPE
VGG	Class 0	0.971	0.857	0.437	0.911	0.900	0.923
VGG	Class 1	0.971	0.857	0.592	0.928	0.897	0.960
My CNN	Class 0	0.961	0.800	0.621	0.895	0.920	0.870
My CNN	Class 1	0.961	0.800	0.380	0.887	0.817	0.957

### Acne classification and the performance of seven-classifier

**Figure 7:**
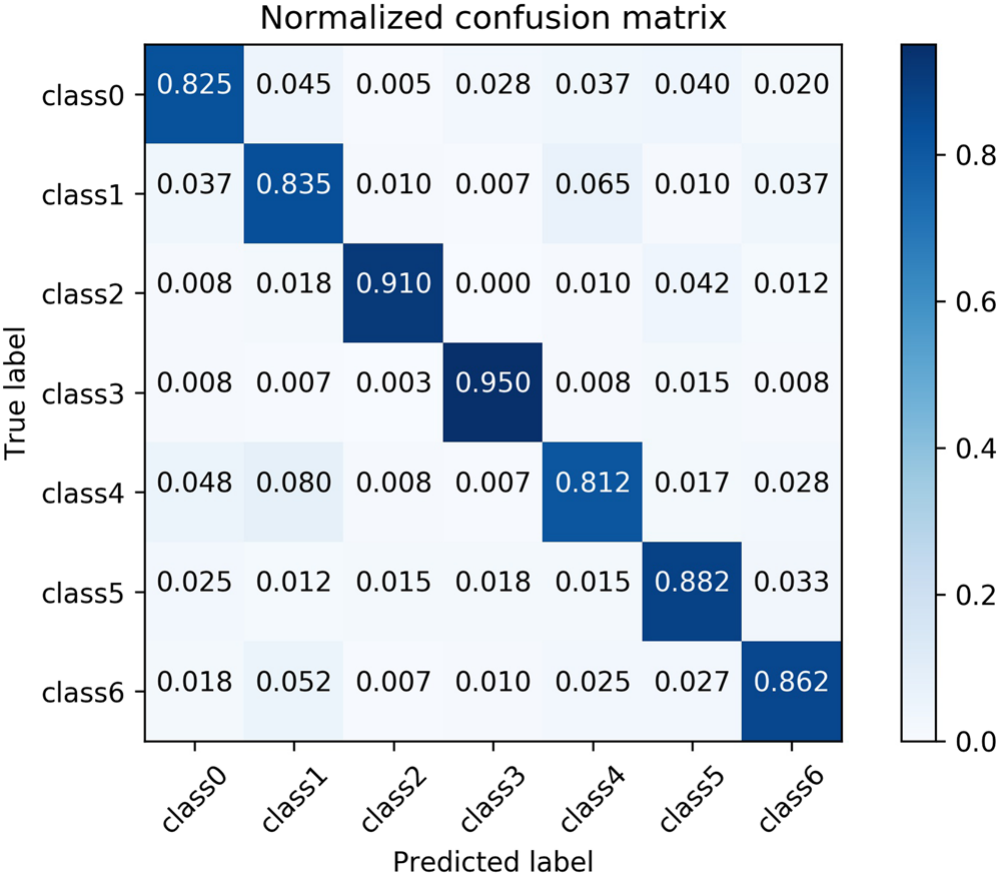
The normalized confusion matrix of the seven-classifier based on VGG16 model (sec. 3.3) Papule (class0), cyst (class1), blackhead (class2), normal skin (class3), pustule (class4), whitehead (class5), nodule (class6). Each column of the matrix represents the instances in a predicted class while each row represents the instances in an actual class.

**Figure 8:**
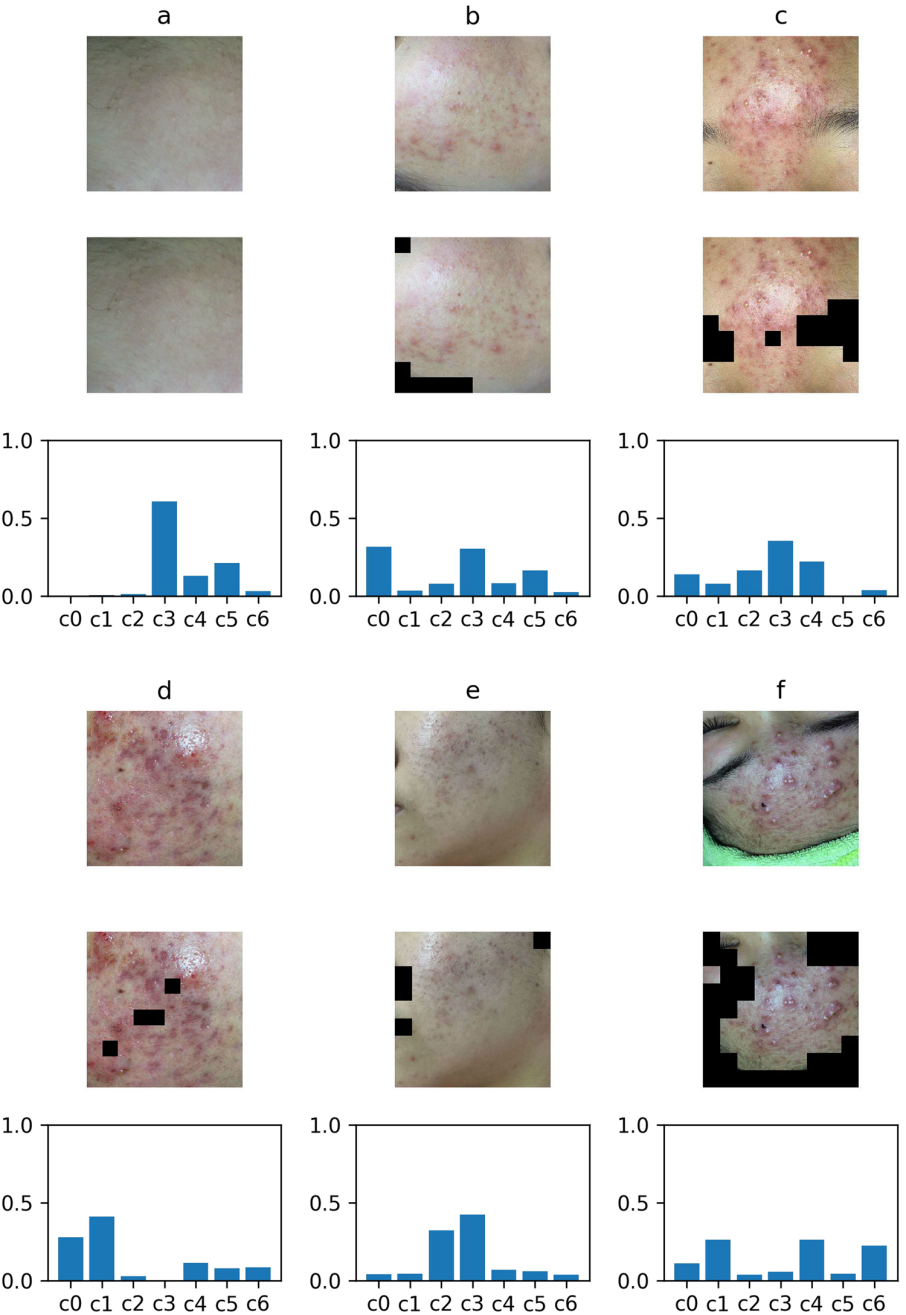
The integral analysis of facial skin area (sec. 3.3) Six images with masks are the input images. The areas with black blocks are non-skin areas, the rest areas are skin areas. The result of integral analysis is shown with the histogram of corresponding proportions. Vertical coordinate of the histogram is the proportion value, abscissa is the type of symptoms, followed by papule (c0), cyst (c1), blackhead (c2), normal skin (c3), pustule (c4), whitehead (c5), nodule (c6).

Because the seven-classification data set is abundant enough after data augmentation, we standardize all the data set with the mean(183.643) and the standard deviation(38.210) of the train data set. When training, we use the categorical cross entropy as the loss function. The categorical cross entropy loss is

7 $$L(X,t)=-\,\sum _{i=0}^{6}\,{t}_{i}\,\mathrm{log}\,{y}_{i},$$

where *y*_*i*_ is the probability that the model assigns to the label *t*_*i*_, and the seven classification label *t* ∈ {0, 1, 2, 3, 4, 5, 6}. And we use the same Adam optimizer whose learning rate is 0.001, beta 1 is 0.9, beta 2 is 0.999 to minimize the loss function, the same batch size, epochs and a random seed. After 50 epochs, we choose the best model by evaluating the performance of the model on seven-classification validation data set. What’s more, we also tune the model with SGD whose learning rate is [0.001, 0.01]. But the performance also doesn’t improve obviously,too. In this paper, the normalized confusion matrix is used to evaluate the performance of seven-classifier based on the pre-trained VGG16 model on the testing data set. The normalized confusion matrix is shown in Fig. 7. The overall description of facial acne symptoms(apply into practice) in this article is shown in Fig. 8. The overall description of the image predicted by our model and the overall description of the experts’ diagnoses are shown in Table 5. By analyzing the normalized confusion matrix, although the model has some wrong classification, it can still complete the classification task well. The accuracy of any class is exceed 81%. And by analyzing the Fig. 8 and the Table 5, it prove the value of the model in practical. In Table 5, we determine the symptoms based on the predicted probability. In particular, We ignore the probability of the normal skin(c3) class, and choose the large predicted probability to determine the symptoms by model. Compare the result of paper(C) with result of experts, our trained model has the ability to diagnose automatically. What’s more, the model can provide an auxiliary diagnosis function.

**Table 5:** The comparisons between the overall description from our method and the description results from experts (sec. 3.3) Papule (c0), cyst (c1), blackhead (c2), normal skin (c3), pustule (c4), whitehead (c5), nodule (c6), P (probability), C (class). The result of the paper(P) is same with the histograms in Fig. 8. The expert’s diagnosis gives the main symptoms of facial vulgaris acne not including normal skin.

Picture	Result of the paper(P)	Result of paper(C)	Result of experts
a	[0.00, 0.00, 0.01, 0.61, 0.13, 0.21, 0.03]	c4, c5	c5
b	[0.32, 0.04, 0.08, 0.30, 0.08, 0.16, 0.02]	c0, c5	c0, c4, c5
c	[0.14, 0.08, 0.16, 0.36, 0.22, 0.00, 0.04]	c0, c2, c4	c2, c4, c5
d	[0.28, 0.41, 0.03, 0.00, 0.11, 0.08, 0.09]	c0, c1, c4	c1
e	[0.04, 0.04, 0.32, 0.42, 0.07, 0.06, 0.04]	c2	c2
f	[0.11, 0.26, 0.04, 0.06, 0.26, 0.04, 0.23]	c1, c4, c6	c4, c5

## Discussion

Compared with traditional skin detection methods based on special color space, the skin detection method in this paper based on skin-and-non-skin binary-classifier is more effective and more robust. Specifically, we find the skin detection method in this paper has a strong anti-interference ability to light conditions and color difference. Therefore, it is more adaptive to many different conditions for the skin detection task. However, it can be seen that there are still some hair areas at junction areas of skin and non-skin that cannot be well detected. The split boundaries in the test images have a clear zigzag shape. It looks bad, but in this experiment, it’s a side effect of an adaptive method, 50 × 50 cutting. And it rarely affects the accuracy of the acne detection in this article. In general, our skin detection method has a better performance in macro aspect, but its micro performance is not satisfying enough.

The seven-type-classifier is the core of the article and is the necessary step to achieve the overall description of facial acne vulgaris. The seven-type-classifier has managed to cut and classify skin areas of the input images. Its practical value is proved through the performance indicators in the Table 5 and Fig. 7. Compared with past methods, we break the restrictions of classification types. We achieve the seven classification of facial acne vulgaris, realize the automatic diagnosis and give the integral analysis of patients’ face. The most important advancement is the number of the classification categories increased to seven, and propose a more detailed holistic analysis.

Generally speaking, if the acne traits are more obvious, the seven-classifier recognition ability is also stronger on this category. Because this type of acne can be better expressed by the 512-dimensional feature vector. In this article, the detection performances of healthy skin, blackhead, whitehead and nodule are better in the automatic diagnosis task, and the detection results of papule, cysts and pustules are relatively poor. The main problem is the 50 × 50 box cannot contain the whole information of cysts and pustules whose disease area is large. The loss of information leads to the effect decline in detection results.

In this paper, we also made a comparison between different binary-classification models. We find the performance of extracting image features by the network we constructed is a little bit weaker than the pre-trained VGG16 model. In the experiment, the ROC was used to evaluate the performance of the two classifiers based on two different CNN models. The experimental results show that the CNN we constructed gains a little bit smaller AUC on the binary-classification test data set, and the detection result is little poorer in the apply task. In seven-classification task, We consider that the small data set and the high similarity of the augmentation data, we use the pre-trained VGG16 model to complete the seven-classification task. So, we use the VGG16 CNN to successfully achieve the transfer learning on small data sets, and to complete the extraction of effective features in the images. The feature vector is regarded as the input of seven-classifier to complete the seven-classification task.

In the future researches, we are prepared to build a more standardized data set, which is supposed to include more types of symptoms and conditions, to meet more standardized training and testing requirements of CNN and the generalization performance. As the data set grows stronger, we will consider to add more detailed description to the automatic diagnosis such as the severe level or the ages of acne vulgaris. In addition, we will consider the use of multiple pre-trained CNN models to replace the current single VGG16 neural network model, to make full use of each neural network model so that we can extract more effective features from the input images.

Furthermore, macro symptoms can be traced back to gene expression. Recent years, many experimental researches have shown that long non-coding RNAs(lncRNAs) and microRNAs(miRNAs) are closely related to human complex diseases. And they could be considered as potential biomarkers for disease diagnosis, treatment and so on^26–28^. Many computational models have been proposed to predict the potential lncRNAs-disease association and miRNAs-disease association^26–31^. These computational models all follow the basic assumption that similar diseases tend to have associations with functionally similar lncRNAs or miRNAs^26–31^. In the future researches, the similarities between facial acne and the diseases in public databases would be computed with the aforementioned computational models to find the associated lncRNAs and miRNAs. It’s obvious that it will help understand the pathogenesis and treatment of facial acne at lncRNA and miRNA level. However, this is also a huge challenge because the known lncRNA-disease associations and the known miRNA-disease associations are rare^27,28^.
